# Monovalent Copper Oxide in Broiler Nutrition: Effects on Performance, Intestinal Lesions, and Oocyst Shedding During Mild *Eimeria* Challenge

**DOI:** 10.3390/vetsci12050494

**Published:** 2025-05-19

**Authors:** Nasima Akter, Thi Hiep Dao, Alip Kumar, David Cadogan, Tamsyn M. Crowley, Amy F. Moss

**Affiliations:** 1 School of Environmental and Rural Science, Faculty of Science, Agriculture, Business and Law, University of New England, Armidale, NSW 2351, Australiatdao2@une.edu.au (T.H.D.); akumar28@une.edu.au (A.K.); 2 Department of Dairy and Poultry Science, Faculty of Veterinary Medicine, Chattogram Veterinary and Animal Sciences University, Chattogram 4225, Bangladesh; 3 Feedworks Australia, P.O. Box 369, Romsey, VIC 3434, Australia; david.cadogan@feedworks.com.au; 4 Institute for Mental and Physical Health and Clinical Translation (IMPACT), School of Medicine, Deakin University, Geelong, VIC 3220, Australia; tamsyn.crowley@une.edu.au

**Keywords:** anticoccidials, broilers, *Eimeria*, lesion score, monovalent copper, oocyst

## Abstract

Coccidiosis is a major economic threat in poultry, and with anticoccidials being phased out, cost-effective alternatives like copper (Cu) supplementation are of interest. This study investigated whether in-feed monovalent Cu (100 ppm) could mitigate the effects of a mild *Eimeria* challenge in broilers. A total of 216 broiler chicks were randomly assigned to three treatments (six replicates, 12 birds/replicate): unchallenged control (NC), challenged control (PC), and challenged + Cu-supplemented. Birds were fed starter (days 1–10), grower (days 10–21), and finisher (days 21–35) diets. On day 14, all birds except the NC group were orally challenged with 5000 oocysts each of *Eimeria acervulina* and *E. maxima*. Feces were collected (days 17–28) for oocyst count, and growth performance, lesion scores (day 21), carcass traits and bone morphology (day 35), gut morphology (days 21 and 35), gizzard weight (days 21 and 35), and cecal bacterial load (days 21 and 35) were evaluated. The mild challenge was confirmed by the observed differences in lesion scores on day 21 (*p* < 0.05), fecal oocyst counts from days 17–28 (*p* < 0.05), and overall mortality (*p* > 0.05) between challenged and unchallenged groups. Copper supplementation tended to improve FCR during the grower phase (1.403 vs. 1.469; *p* = 0.057) and significantly reduced oocyst excretion on days 23–25 (*p* < 0.001) compared to the PC treatment. Although performance benefits were limited, Cu reduced oocyst shedding, indicating potential anticoccidial effects. However, further studies are needed to confirm the consistency of this effect across different doses of Cu in poultry production.

## Simple Summary

Coccidiosis constitutes a significant challenge to the global poultry industry, particularly following the ban on in-feed coccidiostats. Expensive vaccines and economic losses due to coccidiosis make poultry producers seek sustainable alternatives, with copper supplementation being one such alternative. This project explores the effect of dietary copper on mitigating coccidiosis, demonstrating that copper supplementation maintained growth performance and had the potential to reduce the number of *Eimeria* oocysts in the feces of *Eimeria*-challenged birds.

## 1. Introduction

Avian coccidiosis is a parasitic disease that poses a significant threat to the poultry industry worldwide, affecting various species of birds, including chickens and turkeys [1]. This protozoan infection is caused by members of the genus *Eimeria*, with 7 different species (*E. tenella*, *E. necatrix*, *E. brunetti*, *E. maxima*, *E. acervulina*, *E. mitis,* and *E. praecox*) targeting specific regions of the avian intestinal tract [2]. The life cycle of *Eimeria* is complex, involving external sporogony, followed by schizogony and gametogony, which result in oocyst sporulation, shedding, and invasion of host cells, ultimately leading to the destruction of intestinal tissues [3]. *Eimeria* infection leads to reduced productivity, immune suppression, diarrhea, and, in severe cases, mortality, resulting in substantial financial losses each year [4,5]. These losses are primarily driven by reliance on synthetic coccidiostats and the diminished performance of infected birds [4,5]. The disease typically presents as enteritis, with clinical signs including diarrhea, weight loss, poor feed conversion, and impaired growth [4,6,7]. Additionally, sub-clinical *Eimeria* infections are widespread, causing hidden economic losses through decreased feed efficiency and overall flock performance [7,8,9].

Understanding the intricate interactions between *Eimeria* species and the avian host is crucial for developing effective control strategies. Over the years, researchers have made significant strides in the molecular mechanisms underlying coccidiosis pathogenesis, host immune responses, and the development of resistance to anticoccidial drugs [10]. The exploration of novel vaccine candidates and the evaluation of alternative control measures have become key areas of focus to mitigate the impact of avian coccidiosis on poultry production [11]. However, anticoccidial vaccines are expensive and require further development and reduced cost before they can be frequently implemented throughout the broiler industry. Also, three new cryptic *Eimeria* species, named *E. lata, E. nagambie*, and *E. zaria,* have been identified recently that escape immune inhibition from current vaccines [2,12,13].

Moreover, the synthetic coccidiostats commonly used to prevent and manage coccidiosis have been associated with drug residues in poultry products, which may pose serious health risks to consumers and contribute to the growing issue of drug resistance [14]. As a result, coccidiostat-free broiler production, either through legislative or voluntary bans on in-feed coccidiostats, has been adopted in many countries worldwide [15]. Without effective anticoccidial agents, there may be an increase in the prevalence and severity of coccidial infections among poultry. This could lead to economic losses due to reduced growth rates, decreased feed efficiency, and increased mortality [16]. Therefore, any nutritional strategies that may be readily implemented to reduce coccidiosis burden would be of vital importance for both the Australian and global poultry industries. Among the potential candidates as alternatives, copper (Cu) has been explored [17]. Copper is an essential trace element involved in various physiological processes, including enzyme activation, redox reactions, and immune function [18]. The effect of Cu supplementation on *Eimeria*-challenged birds has garnered increasing attention in poultry research due to its potential impact on both disease resistance and birds’ immunity [19]. Copper has been investigated as a potential modulator of the host response to coccidial infection [20]. Recent studies have explored the hypothesis that supplemental Cu may enhance the resilience of broilers challenged with *Eimeria* species by reducing duodenal lesion score [21]. The rationale behind this investigation may lie in the multifaceted roles of Cu in promoting both innate and adaptive immune responses by improving the ileal mucosa-associated bacterial community and intestinal morphology and reducing intraepithelial lymphocytes [22,23].

Copper has been shown to change gut microbiota, regulating the bacterial population in broilers [24], but it is yet unknown if dietary Cu supplementation directly affects intestinal lesions and the *Eimeria* oocysts during mild challenge conditions. Our hypothesis was that the inclusion of Cu may lessen the effects of *Eimeria* challenge and reduce the impact of secondary infection risk in a gut damaged by sub-clinical coccidiosis. Thus, this study was implemented to determine if the in-feed supplementation of monovalent Cu (100 ppm) can assist broilers to lessen the severity of the sub-clinical coccidiosis challenge, in comparison to negative control (unchallenged) and positive control (challenged) groups offered standard industry wheat-soybean meal-based diets. It was hypothesized that Cu supplementation may return performance to that of the negative control treatment.

## 2. Materials and Methods

### 2.1. Experimental Design and Diets

The study was implemented at the Centre of Animal Research and Teaching at the University of New England, Armidale, New South Wales, Australia, approved by its Animal Ethics Committee (Approval number: AEC19-119), and met the requirements of the Australian code of practice to care and use of animals for scientific purposes [25]. A total of 216 one-day-old Ross 308 male broiler chicks were allocated to 18 equal-sized replicate pens (120 × 80 cm) with 12 birds per pen. There were three dietary treatments with six replicate pens (72 birds) per treatment. The initial pen weights of the treatments were statistically comparable. Wood shavings were used as bedding for the birds, which were raised in environmentally controlled rooms to replicate commercial circumstances. Birds had *ad libitum* access to the feed (linear feeder) and water (nipple drinker) throughout the 35-day experiment. Birds had a ‘23-h-on-1-h-off’ lighting regime for the first day and gradually transitioned to an ‘18-h-on-6-h-off’ lighting regime by day 7. An initial room temperature of 33 ± 1 °C was maintained for the first day and gradually decreased to 21 ± 1 °C by the end of the third week, being maintained at this temperature until the end of the experiment. All the rearing conditions were maintained according to the Ross 308 recommendations [26]. Dietary treatments consisted of a negative control (NC) standard industry wheat-soybean meal-based diet without *Eimeria* challenge, a positive control (PC) standard industry wheat-soybean meal-based diet with *Eimeria* challenge, and a Cu-supplemented wheat-soybean meal-based diet (100 ppm of CoRouge, Animine Precision Minerals, Annecy, France) with challenge. The selection of the 100 ppm dose for monovalent Cu supplementation in the Cu-supplemented treatment diet was a strategic choice aimed at balancing efficacy and safety. Research indicates that Cu supplementation at levels exceeding the nutritional requirement (approximately 8 ppm) can improve poultry performance, with 100 ppm being an effective and safe choice [27]. Excessive Cu supplementation (above 125 ppm) has been associated with increased Cu excretion and potential environmental pollution, highlighting the importance of using effective yet environmentally responsible dosages like 100 ppm [28]. Moreover, since our objective was to evaluate the efficacy of Cu supplementation under a sub-clinical or mild *Eimeria* challenge, we deliberately selected the safest effective dose of Cu to avoid potential confounding effects of higher pharmacological levels and to reflect a more conservative and practical approach for industry application. All three diets were initially prepared using the same basal formulation. The Cu diet was then separated and supplemented with monovalent copper at a rate of 100 ppm before pelleting. In contrast, the NC (negative control) and PC (positive control) diets were mixed and pelleted together, as there was no difference in their feed composition. The only distinction between these two treatments was the *Eimeria* challenge: the NC group remained unchallenged, while the PC group received the *Eimeria* challenge. Birds were offered crumbled starter (0–10 days), pelleted grower (10–21 days), and finisher (21–35 days) diets. Feed was pelleted at a temperature of 65 °C. All feeds were formulated to meet the breed’s minimum nutrient requirements [29]. Feedstuffs were analyzed for nutrient content prior to diet formulation. Nutritional contents such as dry matter (DM), apparent metabolizable energy (AME), crude protein (CP), and fat in the primary feed ingredients were analyzed using near-infrared reflectance spectroscopy (Foss NIR 6500, Hillerød, Denmark), standardized with Evonik AMINONIR Advanced calibration, to formulate the experimental diets. The calculated nutritional values of the dietary treatments were used to ensure that all diets met or exceeded the nutrient requirements for broiler chickens as recommended by breeder guidelines. Detailed diet composition and calculated nutrient contents are presented in Table 1. Furthermore, the actual nutrient content of the prepared diets was assessed using standard analytical methods [30], as described in Section 2.6, while Cu content was analyzed following the methodology outlined by Zanu et al. [31]. The analyzed nutrient content of the dietary treatments is reported in Table 2.

### 2.2. Eimeria Challenge and Biosecurity

The birds in each pen were checked for any coccidial infection via fecal oocyst count on the day before the challenge (day 13). On day 14, a mild coccidia challenge was established using *E. acervulina* and *E. maxima* species following procedures previously described by Daneshmand et al. [32] with modifications. The rationale for selecting *Eimeria acervulina* and *Eimeria maxima* in our challenge model was threefold. First, our goal was to induce a mild or sub-clinical infection rather than a severe one. Second, these two species are among the most frequently encountered and economically significant *Eimeria* species in both backyard and commercial broiler operations in Australia [33]. Finally, the distinct difference in oocyst size between these two species also facilitates more accurate differentiation and enumeration of oocysts under microscopic examination [34]. Briefly, the birds in PC and Cu-supplemented treatments (challenged groups) were orally inoculated with 1 mL of live sporulated vaccine of *Eimeria* containing *E*. *acervulina* (5000 oocyst) and *E*. *maxima* (5000 oocyst) from *Eimeria* Pty Ltd. (Ringwood, Victoria, Australia), whereas the birds in the NC group were given 1 mL of sterile phosphate buffer solution (PBS) as a mock treatment on the same day.

The whole housing facility was heat-treated before the arrival of chicks (40 °C for 72 h) to ensure the destruction of any existing coccidia oocysts [35]. Birds in the challenged and unchallenged groups were reared in two separate rooms under the same rearing conditions from day 1 to prevent cross-contamination. These two experimental rooms were identical, as they were part of a single room divided by a heavy curtain for biosecurity purposes. Environmental conditions, including temperature and ventilation, were automatically controlled, regularly monitored (twice a day), and checked to ensure uniformity between both sections. Additionally, all other management practices were consistently applied across the two areas to maintain standardized rearing conditions. Ammonia foot baths were used upon entry and exit of each room. Separate lab coats and footwear were used for each room. All the remaining birds and bedding materials were properly discarded, and the experimental rooms and equipment were cleaned and disinfected accordingly upon finishing the study.

### 2.3. Data and Sample Collection

Body weight (BW) and feed consumption data were recorded on days 10, 21, and 35 of the study. Body weight gain (BWG) and the feed conversion ratio (expressed as feed–gain, FCR) were then calculated using the following formulas:

BWG = Final weight (gm) − Initial weight(gm)

FCR = Total feed intake (gm)/Total body weight gain (gm)

Mortality (number and weight of dead birds) was recorded when it occurred. Feed samples were collected after preparation for nutrient analysis. Fecal sample collection was performed from days 17 to 28 to determine the total *Eimeria* oocyst count. On day 21, 4 sample birds per replicate pen (24 birds/treatment) were randomly collected, weighed, electrically stunned (MEFE CAT 44N, Mitchell Engineering Food Equipment, Clontarf, Queensland, Australia), and euthanized by cervical dislocation for collection of the small intestines to assess lesion score and intestinal morphology (length and diameter). Cecal contents were also collected on day 21 for microbial analyses. On day 35, 4 birds per pen were randomly selected and euthanized using similar procedures to those described for the day 21 sampling. After dissection, the weights of different carcass cuts (breast, thigh and drumstick, and abdominal fat) and gizzard (full and empty) were recorded and expressed as relative weights per unit of live BW. Samples of the small intestine, cecal content, and right tibia were also collected on day 35 to analyze the intestinal morphology, microbiota population, and tibia characteristics, respectively. Lesion scoring was performed in the duodenum, jejunum, and ileum samples by experienced personnel blind to the experiment design. It was performed based on a scale of 0 (none) to 4 (severe lesions with a thickened intestinal wall) following criteria described by Johnson and Reid [36]. Cecal digesta samples were collected and snap-frozen in liquid nitrogen using 2 mL Eppendorf tubes. Then, the samples were kept at −20 °C for further processing. After being collected, the tibial samples were thoroughly defleshed using a scalpel and scissors. A Discover Precision balance (FX-3000i, A & D Company Ltd., Tokyo, Japan) was used to weigh the fresh, wet bones. After three days of air-drying in a fume hood, the bones were reweighed and stored at 5 °C until further analysis.

### 2.4. Feed Analysis

An ultra-centrifugal mill (Retsch ZM 200, Fisher Scientific, Hampton, NH, USA) fitted with a 0.5 mm screen was used to grind the feed materials into fine particles. According to the Dumas combustion method [37], a nitrogen analyzer (LECO Corporation, St. Joseph, MI, USA) was used to determine the protein concentration in the feed, where EDTA was employed as the calibration standard. Using benzoic acid as the calibration standard, the Parr Adiabatic Oxygen Bomb calorimeter (Parr Instrument Co., Moline, IL, USA) was used to measure the gross energy (GE) levels in the feed samples. To determine dry matter (DM), ground feed samples were oven-dried into crucibles for approximately 24 h (to constant weight) at 105 °C, following standard methods [30]. The Cu content of the experimental diets was determined using an inductively coupled plasma-optical emission spectrometry (ICP-OES) instrument (Agilent Technologies, Victoria, Australia) in accordance with the methodology outlined by Zanu et al. [31].

### 2.5. Total Oocyst Count

The fecal samples were collected from days 17 to 28 and aliquoted into three-day pooled samples, such as days 17–19, 20–22, and so on. Since we used a mixed *Eimeria* challenge in this study, and considering the differing prepatent periods of *E. acervulina* (~4 days) and *E. maxima* (5–7 days), we tried to follow recommended fecal collection window of days 4 to 9 post-infection to avoid missing the onset of oocyst shedding [38]. The collected fecal samples were stored at 4 °C both during and after collection, and the pooled samples were then mixed thoroughly. The total oocyst count was performed by the modified McMaster egg-counting technique according to Morris et al. [39] with slight modifications. On the third day of collection, 5 g of the fecal sample was weighed into a 50 mL falcon tube for each. Saturated sodium chloride (NaCl) was added to each of the tubes up to the 50 mL mark and mixed thoroughly to reach a 1:10 dilution. Then, each sample was dispensed into separate McMaster slides using sterile disposable pipettes and allowed to sit for 5 min. The slides were then examined under 100× magnification of the microscope to count and record all oocysts observed. The dilution was employed to obtain a total count of 70 to 220 oocysts per chamber of the slide and adjusted according to the need (higher or lower). The calculation of OPG followed the formula:

$$OPG=\frac{\text{Number of oocyst counted}\times 3.33\times \text{Dilution factor}}{\text{Weight of feces}}$$

### 2.6. Cecal Microbiota Analysis

DNA from the cecal content was extracted using the DNeasy PowerSoil Pro Kit (QIAGEN GmbH, Hilden, Germany), following the manufacturer’s instructions. Then, the specific primers (16S rRNA) were used to determine the relative amounts of *Bacillus* sp., *Bacteroides* sp., *Bifidobacterium* sp., Enterobacteriaceae, *Lactobacillus* sp., *Ruminococcus* sp., and total bacteria (Table 3), expressed as log_10_ genomic DNA copies per gram of cecal digesta as described by Kheravii et al. [40]. Quantitative real-time PCR (Rotorgene 6000 real-time PCR machine, QIAGEN GmbH, Hilden, Germany) was employed to determine the bacterial populations.

### 2.7. Bone Analysis

Using a Kincrome 0–150 mm Digital Vernier caliper (Kincrome, Scoresby, Victoria, Australia), the length (between the tip of the proximal end and the tip of the distal end) and breadth (at the midpoint) of the air-dried tibias were measured. According to Seedor et al. [48], the bone Seedor index was computed as follows: Seedor index = weight of air-dried bone (mg)/length of air-dried bone (mm). To determine the breaking strength of air-dried bones, Instron^®^ electromechanical universal testing equipment (Instron^®^ Mechanical Testing Systems, 825 University Ave., Norwood, MA, USA) was used. The breaking strength was tested with a 3-point flexure test setup at a 300 KN load cell and 50 mm at 0.2 mm/second speed, with 20 data points per second. The data were recorded using the universal materials testing software Bluehill (ver.2, Instron^®^ Mechanical Testing Systems, 825 University Ave., Norwood, MA, USA). The mechanical force at the midpoint of the bone was applied from a 2 cm distance between two fixed points (50 mm) supporting the bone. All the tibias were tested in a single day. Tibia samples were then weighed into crucibles, ashed in a muffle furnace (Carbolite, Sheffield, UK) set to run at 350 °C for 1 h followed by an increase to 600 °C for 13 h and then reweighed. Ash weight was divided by the oven-dried bone weight and multiplied by 100, yielding the ash content (%).

### 2.8. Statistical Analysis

All data analyses were performed using R Commander (version 3.3.1, R Foundation for Statistical Computing, Vienna, Austria). Data were tested for a normal distribution and equal variances between the dietary treatments. A quantile comparison plot was employed to check the data distribution, and then a Levene’s test was used to test the homogeneity of variances between the treatments. Depending on the results produced by the above 2 tests, either a one-way ANOVA or the non-parametric Kruskal–Wallis test was used to test statistical differences between the treatments. In the present study, we employed the non-parametric Kruskal–Wallis test to analyze the intestinal lesion score and OPG count data, while other parameters were tested using the one-way ANOVA. Tukey’s post hoc test was employed to identify pairwise differences between the treatments from significant ANOVA results. *p*-values were considered significant at ≤0.05, while values between 0.05 and 0.10 were considered to indicate a trending pattern.

## 3. Results

### 3.1. Growth Performance

The growth performances of experimental treatments in the starter, grower, finisher, and overall periods are shown in Table 4. In the starter phase, production performance data were presented and analyzed for only two groups (the control group and the Cu-supplemented group) since no *Eimeria* challenge was introduced during this phase. For the subsequent phases, data were categorized into three groups (NC, PC, and Cu-supplemented) and analyzed accordingly, as these phases involved an *Eimeria* challenge. In the starter phase, there were no significant differences between weight gain, FI, or FCR between treatments. Although the difference was not statistically significant, the challenge had the greatest impact on growth performance during the finisher phase, where the FCR of the PC treatment tended to worsen compared to the NC treatment (*p* = 0.071, Table 4), likely due to comparatively higher feed intake and lower weight gain. Copper supplementation for the challenged birds tended to increase the feed efficiency in the grower phase compared to the PC treatment (*p* = 0.057, Table 4). However, this effect was not observed during the finisher phase and overall period (Table 4). Mortality remained similar across all treatments throughout the study period and was within an acceptable range (*p* > 0.05, Table 4).

### 3.2. Intestinal Lesion Score and Morphology

**Figure 1:**
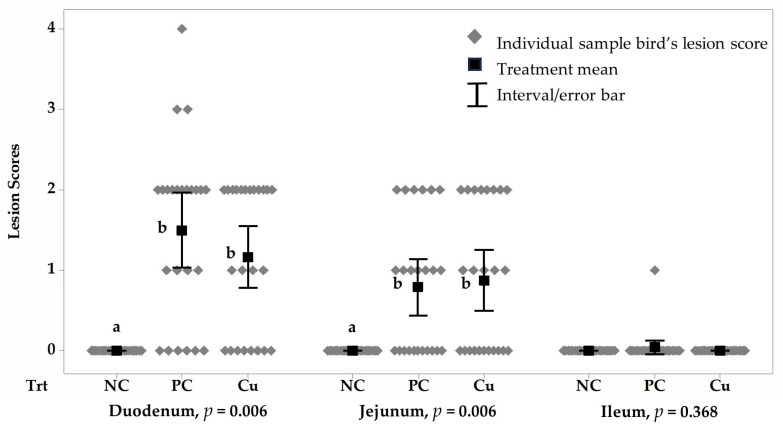
Intestinal lesion scores of negative control (NC), *Eimeria*-challenged positive control (PC), and *Eimeria*-challenged Cu-supplemented broilers on day 21. (^a,b^ Means between treatments of different parts of small intestine not sharing a common suffix are significantly different at the 5% level of probability, and interval/error bars represent standard deviations of the means).

The intestinal lesion score results indicated that birds in the PC and Cu-supplemented groups exhibited significantly higher duodenal (*p* = 0.006) and jejunal (*p* = 0.006) lesion scores compared to the NC treatment, which had a score of zero on day 21 (Figure 1), while the ileal lesion scores remain comparable between treatments on day 21 (*p* = 0.368, Figure 1).

The results on intestinal length and diameter on days 21 and 35 are given in Table 5. Birds in the PC treatment group had higher jejunal and ileal length and diameter compared to the NC treatment group on day 21 (*p* < 0.05, Table 5). Similarly, higher duodenal, jejunal, and ileal lengths were observed in birds in the PC treatment group compared to the NC treatment group on day 35 (*p* ≤ 0.001; Table 5). Copper supplementation to the challenged birds did not affect intestinal length and diameter on day 21, but increased ileal length compared to the PC treatment on day 35 (*p* < 0.001; Table 5).

### 3.3. Fecal Oocyst Count

**Figure 2:**
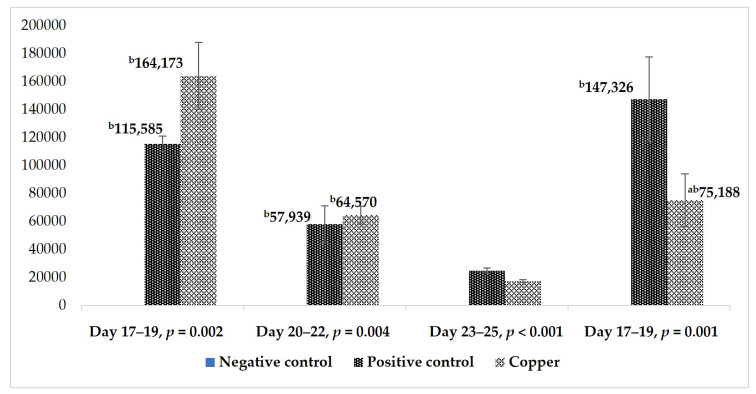
Total oocyst count per gram of feces of broilers of different treatments from days 17 to 28 (^a,b^ Means between treatments of different day groups not sharing a common suffix are significantly different at the 5% level of probability and and error bars present standard errors of the means).

The results of total *Eimeria* oocyst counts from days 17 to 28 are presented in Figure 2. The oocyst counts in the NC treatment remained low (nil) throughout the feces collection period compared to the PC and Cu-supplemented group (*p* < 0.05, Figure 2), as expected, since these birds were not subjected to an *Eimeria* challenge. Birds in the challenged group exhibited positive oocyst shedding from days 17 to 28 (Figure 2). The results showed that Cu supplementation significantly reduced OPG counts compared to the PC group from days 23 to 25 (*p* < 0.001, Figure 2). Though statistical significance was not obtained, the OPG count tended to be lower (by 49%) in the Cu-treated group compared to the PC group (147,326 vs. 75,188) from days 26 to 28 (Figure 2) and shifted toward the NC group (*p* > 0.05), with no significant difference between the Cu and NC groups.

### 3.4. Carcass Yield and Gizzard Weight

The relative carcass yield on day 35 and the relative weights of full and empty gizzards on days 21 and 35 of experimental treatments are shown in Table 6 and Table 7, respectively. The *Eimeria* challenge significantly reduced the relative breast weight on day 35 (*p* = 0.006, Table 6), while it had no effect on the relative weights of the thigh, drumstick, or abdominal fat (*p* > 0.05, Table 6). Copper supplementation in challenged birds did not significantly influence any carcass yield parameters, including breast, thigh, drumstick, and abdominal fat weights, compared to the PC treatment (*p* > 0.05, Table 6). However, Cu supplementation significantly reduced the weight of the empty gizzard compared to the NC treatment on day 21 (*p* = 0.037, Table 7), while full gizzard weight remained unaffected (*p* > 0.05, Table 7). This effect was not observed on day 35, as both full and empty gizzard weights showed no significant differences among treatments (*p* > 0.05, Table 7).

### 3.5. Cecal Microbiota Profile

The cecal microbiota populations of the treatment groups on days 21 and 35 are shown in Table 8. Copper supplementation in the challenged birds reduced the numbers of *Bacillus* sp. compared to the PC group on day 21 (*p* = 0.010, Table 8). A decreasing trend was also observed in *Bifidobacterium* sp. counts in the Cu-supplemented group relative to the PC group, although this was not statistically significant (*p* = 0.073; Table 8). The other microbiota populations were not different between the treatments on days 21 and 35 (*p* > 0.05, Table 8).

### 3.6. Tibia Characteristics

The tibia morphology and ash content of the experimental treatments on day 35 are shown in Table 9. The results showed that tibia weight, length, diameter, breaking strength, and ash content were not different between the dietary treatments on day 35 (*p* > 0.05; Table 9).

## 4. Discussion

The results related to growth performance post-challenge, low mortality rates, intestinal morphology, intestinal lesion scores, and fecal *Eimeria* oocyst counts confirmed that the negative control (NC) group remained free of coccidiosis. In contrast, the challenged groups exhibited clear signs of infection. These findings indicate that a mild *Eimeria* challenge was successfully established in the current study. In the present study, the oral *Eimeria* challenge (PC) resulted in a 3.0% reduction in BWG compared to the birds on the negative control treatment during the finisher period across days 21–35 (1399 vs. 1443 g). Furthermore, challenged birds showed a 5.9% increase in FCR from days 1 to 35 compared to the unchallenged birds (1.62 vs. 1.53). Coccidiosis infection destroys host mucosal cells, resulting in nutrient leakage, impaired digestion, and absorption, which compromises the overall productivity of chickens [49,50]. The results of the current study are consistent with the results of other researchers who observed a performance reduction in broiler chickens after a challenge by *Eimeria* sp. [21,51,52].

The growth performance of broilers under *Eimeria* challenge in the present study was not improved by the dietary supplementation of Cu. Zaghari et al. [53] observed a similar effect with Cu supplementation of up to 150 ppm. Findings from other studies demonstrated that higher doses of Cu can enhance growth performance in broilers [24,54,55]. As opposed to the present study, these studies obtained better growth performance by adding Cu at higher dose rates (>100 ppm). Thus, the limited effects of Cu supplementation in the present study might be due to the low dosage (which was chosen to mitigate environmental concerns), which may not be sufficient to reduce the deleterious effects caused by the infection. Nevertheless, conflicting findings have been reported in the literature regarding the supplementation of Cu in broiler diets, which might be attributed to several factors such as diet composition, gastrointestinal status, and interactions between these factors [53,56,57,58].

*Eimeria* species in poultry cause site-specific infections within the gastrointestinal tract, with each species targeting a distinct intestinal region [59]. This localization is a key factor in diagnosing coccidiosis, as lesion patterns correspond to the specific *Eimeria* involved. The *Eimeria* species used for the challenge in the present study—*E. acervulina* and *E. maxima*—have a predilection for the small intestine, with *E. acervulina* primarily infecting the upper small intestine (duodenum) and *E. maxima* targeting the mid-section (jejunum) [59]. A previous study showed that Cu supplementation at 150 ppm can significantly reduce the duodenal lesion score in *Eimeria*-challenged broilers while the ileal and jejunal scores remain unchanged [21]. This finding partially supports the result of the present study, where Cu supplementation did not reduce lesion scores in the ileum and jejunum of broilers. Anissimova et al. [60] showed that dietary supplementation of Cu at 400 ppm could reduce cecal lesion scores. Differences in intestinal sections and/or Cu dosages could be responsible for the variations between studies.

The present study demonstrated that the *Eimeria* challenge resulted in an increased length and diameter of the jejunum and ileum regardless of Cu supplementation in the diet. An increase in intestinal length in broilers under *Eimeria* infection could be associated with the hypertrophy of the intestinal mucosa, which might be a response to the parasitic challenge [61]. The increase in length may also be a compensatory mechanism related to the need for greater absorptive surface area to counteract the effects of damaged intestinal villi [62]. However, the efficiency of nutrient absorption may still be compromised due to the altered structure and function of the intestinal mucosa. *Eimeria* infection can induce mucosal hyperplasia, characterized by the proliferation of epithelial cells in the intestinal lining. This hyperplasia is an adaptive response to the destruction of intestinal villi caused by coccidial invasion. The increased cell turnover may contribute to changes in the overall length of the intestine [63]. As high energy levels are required for maintaining intestinal homeostasis [64], the longer intestine in *Eimeria*-challenged birds might partly explain the lower growth performance observed in the respective treatments.

In addition to antibacterial [65] and antiviral properties [66], Cu can effectively reduce the number of *Eimeria* oocytes excreted through feces. Our theory of mitigating coccidiosis through Cu supplementation is supported by the results of the present study, where broilers fed Cu-supplemented diets (100 mg/kg) had fewer discharged oocysts than the PC group on days 23–28. The present findings substantiate previous reports, where the number of fecal oocysts was reduced eight times in broilers fed a diet supplemented with Cu and Zn [53]. It has been suggested that Cu supplementation has the potential to raise IgA levels, which may strengthen intestinal defense against *Eimeria* by binding directly to the oocysts’ surface and preventing them from adhering to the intestinal epithelium [20,67,68]. Copper ions may also increase the permeability of the oocyte membrane, which facilitates its destruction [69,70]. Reducing the number of oocysts released into the environment through feces could be very helpful in reducing reinfection, especially in subsequent poultry flocks through soil, dust, or reused litter [71]. Therefore, further investigation into the effects of Cu supplementation in poultry diets on the environmental accumulation of coccidia oocysts across multiple flock cycles would be valuable. However, variations were observed in the oocyst-counting data in the earlier days of infection in the current study, though these differences were not statistically significant. This variability may reflect the dynamic nature of parasite–host interactions and highlight the need for additional research to better understand the consistency of Cu’s effects, optimize its dosage and application, and further explore its mechanism of action in controlling sub-clinical *Eimeria* infections more effectively.

Many studies have reported that infection with *E. acervulina* and *E. maxima* reduced nutrient digestibility, especially amino acid (AA) digestibility, in broiler chickens [72,73]. Reduced AA digestibility may lead to decreased meat yield because AAs are building blocks for proteins in meat [74,75,76]. The *E. acervulina* and *E. maxima* species are also demonstrated to deteriorate the quality of breast meat [77]. Thus, the current findings were consistent with those previously reported in the literature. The proliferation of *Eimeria* in the intestines of challenged birds might impair gut osmolality and reduce glucose, sodium, and potassium absorption, resulting in reduced protein synthesis and, subsequently, a worsened carcass yield, as shown in the present study and others [76,78,79]. Alternatively, Philpot et al. [80] reported that a higher dose (270 ppm) of dietary Cu supplementation in healthy birds improved the total breast yield of broilers at 53 days old. It is possible that Cu supplementation at 100 ppm could not overcome the deleterious effect of the *Eimeria* challenge to yield a better carcass weight in the present study. The reason for the lower gizzard weight in challenged birds supplemented with Cu compared to the NC unchallenged group is unclear.

*Bifidobacteria* sp., *Lactobacillus* sp., *Bacillus* sp., and *Bacteroides* sp. are generally considered beneficial gut bacteria [81,82,83]. In the present study, their counts remained largely unchanged across treatments, suggesting that the sub-clinical *Eimeria* challenge did not markedly disrupt the gut microbial community. However, the number of cecal *Bacillus* sp. was significantly reduced in the Cu-supplemented group compared to the PC group, possibly due to the known antibacterial properties of Cu [65]. Previous studies have reported increased *Lactobacillus* counts in birds challenged with *Eimeria* [84,85], potentially due to higher protein levels and increased mucus secretion from damaged intestinal cells, which provide substrates for microbial growth [86,87]. Conversely, some studies have shown no effect of coccidial vaccination on *Bacteroides* and *Bacillus* populations [88]. Taken together, these findings suggest that while a strong *Eimeria* challenge may disrupt gut microbiota, sub-clinical infections may not, and nutritional strategies such as Cu supplementation may selectively reduce certain microbial populations like *Bacillus* sp. Negative correlations between dietary Cu and populations of both beneficial and pathogenic bacteria, including *Lactobacillus*, *Bacteroides*, and Enterobacteriaceae, have been previously documented [89].

It is also reported that antibiotic Apramycin treatment increased the numbers of *Bacteroides* sp. and *Escherichia coli* while reducing the numbers of *Bifidobacteria* sp. and *lactobacillus* sp. in the caeca [90]. Rosen [91] added that growth-promoting antibiotics reduced the number and activity of gut microbiota. Collectively, the findings of this study and others suggest that increased dietary Cu levels might result in a decrease in the number of certain bacteria in the gut, which may be similar to the effects of antibiotic growth promoters.

The long bones—such as the tibia—play a pivotal role in bearing the birds’ body weight and reinforcing the integrity of the musculoskeletal framework [92]. Previous studies have indicated that an *Eimeria* challenge might adversely affect bone health as it impairs the duodenum and upper jejunum—the main sites of mineral absorption [93,94]. Similarly, Shaw et al. [95] observed that a *Eimeria* challenge decreased growth performance and the absorption of calcium and phosphorus, resulting in reduced bone breaking strength in birds. Mireles et al. [96] hypothesized that the release of tumor necrosis factor (TNF-α), IL-1, and IL-9 increased bone resorption in *Eimeria*-challenged birds, which may consequently reduce bone quality parameters. Banks et al. [97] and Abdullah et al. [98] observed improved tibia morphology in healthy broiler chickens fed diets supplemented with Cu. According to the results of the current study, neither coccidiosis nor dietary Cu supplementation affects bone quality in broiler chickens. Mild *Eimeria* challenge could be a reason for uncompromised bone quality in the current study.

In the present study, the authors initially hypothesized that Cu supplementation could mitigate the effects of sub-clinical coccidiosis and restore broiler performance to levels comparable to the unchallenged negative control group. However, our findings did not show significant differences in performance metrics among the NC, PC, and Cu-supplemented groups. The primary observed effect of Cu supplementation was a reduction in oocyst excretion at specific time points; however, the results were inconsistent, potentially due to factors such as the variability in parasite–host interactions, the type of challenge, the Cu dosage, and the *Eimeria* species used. Therefore, while Cu showed some potential anticoccidial effects, the hypothesis was not fully supported, and further studies are necessary to validate these findings.

## 5. Conclusions

This study demonstrates the potential of Cu supplementation to reduce oocyst shedding in broiler chickens challenged with a mild *Eimeria* infection. Although a significant reduction in oocyst count was observed at three specific time points, substantial variation remained within the data. This variability may reflect the complex and dynamic nature of parasite–host interactions and suggest that further research is necessary to better understand the consistency of Cu’s effects, optimize its dosage and application, and explore its mechanism of action in controlling sub-clinical *Eimeria* infections more effectively.

## Figures and Tables

**Table 1:** Diet composition and calculated nutrient values of basal diets ^1^ (as-fed basis).

Ingredients, %	Starter (Days 0–10)	Grower (Days 10–21)	Finisher (Days 21–35)
Soybean meal	33.47	25.88	20.76
Canola oil	2.89	4.41	5.18
Wheat	60.14	61.75	66.48
Canola meal	0.00	5.00	5.00
Limestone	1.52	1.35	1.24
Salt	0.173	0.185	0.193
Mono-dicalcium phosphate	0.562	0.334	0.137
Sodium bicarbonate	0.130	0.112	0.097
L-lysine	0.275	0.258	0.249
DL-methionine	0.336	0.264	0.233
L-threonine	0.135	0.097	0.077
L-valine	0.011	0.000	0.000
Choline chloride 75%	0.025	0.025	0.020
^2^ Vitamin and mineral premix	0.175	0.175	0.175
Filler (bentonite)	0.100	0.100	0.100
^3^ Xylanase	0.025	0.025	0.025
^4^ Phytase	0.030	0.030	0.030
Total	100	100	100
**Calculated nutrient, % (Otherwise, as stated)**			
Dry matter	90.5	90.6	90.6
AME (Kcal/kg)	3005	3109	3198
Crude protein	23.39	21.77	19.84
Cude fibre	2.62	2.93	2.84
Crude fat	4.17	5.75	6.53
Ash	5.14	4.62	4.07
^5^ Dig lysine	1.28	1.150	1.020
Dig methionine	0.623	0.549	0.497
Dig methionine + cystine	0.950	0.870	0.800
Dig cysteine	0.323	0.316	0.298
Dig threonine	0.860	0.770	0.680
Dig tryptophan	0.300	0.278	0.253
Dig glycine	0.778	0.739	0.671
Dig arginine	1.370	1.230	1.090
Dig serine	0.778	0.739	0.671
Dig histidine	0.507	0.466	0.419
Dig isoleucine	0.877	0.796	0.710
Dig leucine	1.465	1.343	1.207
Dig phenylalanine	0.979	0.893	0.804
Dig tyrosine	0.823	0.743	0.672
Dig valine	0.960	0.882	0.796
Calcium	0.960	0.870	0.780
Available phosphorus	0.480	0.435	0.390
Sodium	0.160	0.160	0.160
Chloride	0.230	0.230	0.230
Potassium	0.944	0.856	0.767
Linoleic acid	1.215	1.422	1.550
Choline (mg/kg)	1867	1963	1810
Dietary electrolyte balance (mEq/kg)	246	224	201

^1^ The copper treatments were created by adding Cu into the basal diets at the expense of the filler (bentonite). ^2^ Vitamin and mineral premix per kg diet: vitamin A, 12 MIU; vitamin D, 5 MIU; vitamin E, 75 mg; vitamin K, 3 mg; nicotinic acid, 55 mg; pantothenic acid, 13 mg; folic acid, 2 mg; riboflavin, 8 mg; cyanocobalamin, 0.016 mg; biotin, 0.25 mg; pyridoxine, 5 mg; thiamine, 3 mg; antioxidant, 50 mg; Cu, 16 mg as copper sulfate; Mn, 60 mg as manganese sulfate; Mn, 60 mg as manganous oxide; I, 0.125 mg as potassium iodide; Se, 0.3 mg; Fe, 40 mg, as iron sulfate; Zn, 50 mg as zinc oxide; Zn, 50 mg as zinc sulfate. ^3^ Xylanase 8000 L, Danisco Animal Nutrition (IFF), Oegstgeest, The Netherlands. ^4^ Axtra PHY 5000L, Danisco Animal Nutrition (IFF), Oegstgeest, The Netherlands. ^5^ Digestible amino acid coefficients for raw ingredients were determined by Near-Infra Red spectroscopy (Foss NIR 6500, Hillerød, Denmark) standardized with Evonik AMINONIR^®^ Advanced calibration.

**Table 2:** Analyzed nutrient values of experimental diets (as-is basis).

Feeding Phase	Treatment	Dry Matter (%)	Gross Energy (kcal/kg)	Crude Protein (%)	Cu (mg/kg)
Starter	Control	87.08	3948	22.52	17.95
	Copper	86.56	3946	23.34	84.03
Grower	Control	86.47	4008	21.17	17.39
	Copper	86.83	4006	20.73	90.12
Finisher	Control	87.14	4065	20.74	16.48
	Copper	86.72	4061	20.18	113.23

**Table 3:** The sequence of primers used for the qPCR analysis of selected bacterial populations in cecal content.

Target Group or Organism	Primer Sequence (5′–3′)	Annealing Temperature (°C)	Reference
*Bacillus* sp.	F-GCA ACG AGC GCA ACC CTT GA R-TCA TCC CCA CCT TCC TCC GGT	63	Abou-Elkhair et al. [41]
*Bacteroides* sp.	F-GAG AGG AAG GTC CCC CAC R-CGC TAC TTG GCT GGT TCA G	63	Layton et al. [42]
*Bifidobacterium* sp.	F-GCG TCC GCT GTG GGC R-CTT CTC CGG CAT GGT GTT G	63	Requena et al. [43]
Enterobacteriaceae	F-CAT TGA CGT TAC CCG CAG AAG AAG C R-CTC TAC GAG ACT CAA GCT TGC	63	Bartosch et al. [44]
*Lactobacillus* sp.	F-CAC CGC TAC ACA TGG AG R-AGC AGT AGG GAA TCT TCC A	63	Wise and Siragusa [45]
*Ruminococcus* sp.	F-GGC GGC YTR CTG GGC TTT R-CCA GGT GGA TWA CTT ATT GTG TTA A	63	Ramirez-Farias et al. [46]
Total bacteria	F-CGG YCC AGA CTC CTA CGG G R-TTA CCG CGG CTG CTG GCA C	63	Lee et al. [47]

**Table 4:** Growth performance of broilers of experimental treatments during the study.

Treatment		Weight Gain (g)	Feed Intake (g)	FCR	Mortality (%)
Starter	Control	258	352	1.367	0.00
	Copper	254	345	1.362	0.00
	^1^ SEM	4.12	7.17	0.03	0.00
	*p*-value	0.548	0.508	0.904	1.00
Grower	Negative control	739	1079	1.463	1.39
	Positive control	747	1096	1.469	1.39
	Copper	752	1055	1.403	1.39
	SEM	12.50	12.35	0.02	1.38
	*p*-value	0.746	0.095	0.057	1.00
Finisher	Negative control	1443	2315	1.606	2.78
	Positive control	1399	2422	1.735	0.00
	Copper	1400	2421	1.730	1.39
	SEM	16.43	26.69	0.03	1.29
	*p*-value	0.498	0.189	0.071	0.342
Overall	Negative control	2431	3721	1.532	4.17
	Positive control	2402	3887	1.620	2.78
	Copper	2403	3871	1.610	2.77
	SEM	18.67	35.74	0.02	2.18
	*p*-value	0.807	0.125	0.085	0.874

^1^ SEM = Standard error of mean.

**Table 5:** Intestinal length and diameter of broilers of experimental treatments on day 21 and day 35 (cm).

Treatment	Duodenum		Jejunum		Ileum	
	Length	Diameter	Length	Diameter	Length	Diameter
**Day 21**						
Negative control	26.1	1.83	^a^ 58.8	^a^ 1.90	^a^ 60.5	^a^ 1.59
Positive control	27.0	1.80	^b^ 67.8	^b^ 2.23	^b^ 75.0	^b^ 1.79
Copper	27.3	1.82	^b^ 67.7	^ab^ 2.15	^b^ 70.7	^b^ 1.81
SEM	0.52	0.10	1.46	0.08	1.52	0.04
*p*-value	0.290	0.977	<0.001	0.029	<0.001	0.005
**Day 35**						
Negative control	^a^ 28.5	2.30	^a^ 68.0	2.27	^a^ 68.9	1.89
Positive control	^b^ 31.3	2.25	^b^ 80.7	2.50	^b^ 75.6	1.92
Copper	^b^ 32.9	2.36	^b^ 77.0	2.38	^c^ 82.1	2.05
SEM	0.66	0.06	1.68	0.09	1.70	0.05
*p*-value	0.001	0.516	<0.001	0.210	<0.001	0.098

^a,b,c^ Means within columns not sharing a common suffix are significantly different at the 5% level of probability.

**Table 6:** Relative carcass yield of broiler birds of different treatments on day 35 (g/kg body weight).

Treatment	Breast	Thigh	Drumstick	Abdominal Fat
Negative control	^b^ 179	101	87.0	10.43
Positive control	^a^ 173	101	87.1	9.14
Copper	^a^ 171	103	87.2	11.04
SEM	1.60	1.27	0.84	0.62
*p*-value	0.006	0.430	0.992	0.123

^a,b^ Means within columns not sharing a common suffix are significantly different at the 5% level of probability.

**Table 7:** Relative weights of full and empty gizzards of broilers of different treatments on days 21 and 35 (g/kg body weight).

Treatment	Day 21		Day 35	
	Gizzard Full	Gizzard Empty	Gizzard Full	Gizzard Empty
Negative control	25.0	^b^ 19.0	16.6	12.6
Positive control	27.1	^ab^ 18.4	16.8	12.6
Copper	24.7	^a^ 17.0	17.3	12.7
SEM	0.81	0.51	0.70	0.38
*p*-value	0.098	0.037	0.751	0.996

^a,b^ Means within columns not sharing a common suffix are significantly different at the 5% level of probability.

**Table 8:** Cecal microbiota of broiler chickens of different treatments on day 21 and day 35 (log_10_ [genomic DNA copies/g of cecal contents]) content).

Treatment	*Lactobacillus* sp.	*Ruminococcus* sp.	*Bacteroides* sp.	*Bacillus* sp.	*Bifidobacterium* sp.	Enterobacteriaceae	Total Bacteria
**Day 21**							
Negative control	9.18	10.09	7.70	^ab^ 8.32	9.31	8.96	11.36
Positive control	9.33	10.14	7.59	^b^ 8.54	9.74	8.84	11.45
Copper	9.35	10.08	7.56	^a^ 7.98	9.13	8.89	11.44
SEM	0.04	0.02	0.03	0.08	0.11	0.06	0.02
*p*-value	0.119	0.630	0.106	0.010	0.073	0.796	0.238
**Day 35**							
Negative control	9.15	10.00	7.62	8.65	10.28	8.66	11.44
Positive control	9.17	9.99	7.51	8.47	10.30	8.70	11.45
Copper	9.17	9.95	7.52	8.66	10.36	8.62	11.45
SEM	0.02	0.02	0.03	0.04	0.05	0.07	0.02
*p*-value	0.943	0.391	0.160	0.098	0.853	0.884	0.977

^a,b^ Means within columns not sharing a common suffix are significantly different at the 5% level of probability.

**Table 9:** Tibia morphology and ash content of broilers of different experimental treatments on day 35.

Treatment	Fresh Weight (g)	Air-Dry Weight (g)	Length (mm)	Seedor Index	Diameter (mm)	Breaking Strength (N)	Ash as Is (%)
Negative control	13.1	7.01	92.2	75.8	7.97	400	39.8
Positive control	11.9	6.23	91.8	70.5	7.13	371	38.9
Copper	12.9	6.82	91.8	74.3	7.34	405	40.1
SEM	0.60	0.32	0.62	3.34	0.38	21.56	0.42
*p*-value	0.380	0.282	0.827	0.534	0.301	0.494	0.188

## Data Availability

The research data supporting this study will be shared upon reasonable request made to the corresponding author. The data are not publicly available due to privacy concerns.
